# Smart city energy efficient data privacy preservation protocol based on biometrics and fuzzy commitment scheme

**DOI:** 10.1038/s41598-024-67064-z

**Published:** 2024-07-13

**Authors:** Vincent Omollo Nyangaresi, Zaid Ameen Abduljabbar, Keyan Abdul-Aziz Mutlaq, Salim Sabah Bulbul, Junchao Ma, Abdulla J. Y. Aldarwish, Dhafer G. Honi, Mustafa A. Al Sibahee, Husam A. Neamah

**Affiliations:** 1 Department of Computer Science and Software Engineering, Jaramogi Oginga Odinga University of Science and Technology, Bondo, 40601 Kenya; 2 Department of Applied Electronics, Saveetha School of Engineering, SIMATS, Chennai, 602105 Tamilnadu India; 3 Department of Computer Science, College of Education for Pure Sciences, University of Basrah, Basrah, 61004 Iraq; 4 College of Big Data and Internet, Shenzhen Technology University, Shenzhen, 518118 China; 5 Shenzhen Institute, Huazhong University of Science and Technology, Shenzhen, 518000 China; 6 IT and Communications Center, University of Basrah, Basrah, 61004 Iraq; 7 School of Computer Sciences, UniversitiSains Malaysia, USM, 11800 Gelugor, Penang Malaysia; 8 Directorate General of Education Basra, Ministry of Education, Basra, 61004 Iraq; 9 Department of IT, University of Debrecen, Debrecen, 4002 Hungary; 10 National Engineering Laboratory for Big Data System Computing Technology, Shenzhen University, Shenzhen, 518060 China; 11 Computer Technology Engineering Department, Iraq University College, Basrah, 61004 Iraq; 12 Mechatronics Department, Faculty of Engineering, University of Debrecen, Ótemető U. 4-5, Debrecen, 4028 Hungary

**Keywords:** Authentication, Biometrics, Fuzzy commitment, Security, Privacy, Efficiency, Hamming distance, Smart city, Electrical and electronic engineering, Energy infrastructure

## Abstract

Advancements in cloud computing, flying ad-hoc networks, wireless sensor networks, artificial intelligence, big data, 5th generation mobile network and internet of things have led to the development of smart cities. Owing to their massive interconnectedness, high volumes of data are collected and exchanged over the public internet. Therefore, the exchanged messages are susceptible to numerous security and privacy threats across these open public channels. Although many security techniques have been designed to address this issue, most of them are still vulnerable to attacks while some deploy computationally extensive cryptographic operations such as bilinear pairings and blockchain. In this paper, we leverage on biometrics, error correction codes and fuzzy commitment schemes to develop a secure and energy efficient authentication scheme for the smart cities. This is informed by the fact that biometric data is cumbersome to reproduce and hence attacks such as side-channeling are thwarted. We formally analyze the security of our protocol using the Burrows–Abadi–Needham logic logic, which shows that our scheme achieves strong mutual authentication among the communicating entities. The semantic analysis of our protocol shows that it mitigates attacks such as de-synchronization, eavesdropping, session hijacking, forgery and side-channeling. In addition, its formal security analysis demonstrates that it is secure under the Canetti and Krawczyk attack model. In terms of performance, our scheme is shown to reduce the computation overheads by 20.7% and hence is the most efficient among the state-of-the-art protocols.

## Introduction

A smart city refers to a geographical area where technologies such as energy production, logistics and information communication technology are amalgamated to enhance environmental quality, intelligent development, citizen well-being, participation and inclusion. As explained in^1,2^, smart cities utilize data-driven technologies to boost sustainability, efficiency, quality of life of the citizens and streamline city services. In addition, the usage of smart city data and technologies facilitate efficient and optimized management of resources, urban services and assets, as well as aiding in making informed decisions^3,4^. The advancements in big data, cloud computing, Flying Ad-Hoc Networks (FANET), Wireless Sensor Networks (WSNs), Artificial Intelligence (AI), 5th generation mobile network (5G) and Internet of Things (IoT) have led to considerable traction towards smart cities^5–8^. These technologies enable smart cities to collect, analyze and share data from a myriad of sources such as social media, sensors, vehicles, electronic devices, machines and mobile devices. The capabilities of interconnecting a large pool of heterogeneous smart devices enable seamless connections to the smart city environment devoid of communication loss^9^. This helps improve smart city operations and services in terms of enhanced traffic flow, reduced crime rates, energy efficiency and improved citizen engagement.

According to^10^, the deployment of heterogeneous communication modes to interconnect smart devices enables the smart cities to have direct exploitation of resources, facilitating easy access to information. In addition, it offers pervasive computing, comprehensive perception, ubiquitous and reliable services. These services may include smart parking, environmental monitoring^11^, smart traffic lights, rescue operations^12^, smart transportation, remote health monitoring, surveillance, disaster management, search, and traffic monitoring, which can be accomplished by WSNs or Internet of Drones (IoD). As such, smart cities are characterized by high responsiveness, high connectivity, enhanced sustainability, improved quality of life, elevated intelligence, enhanced resource utilization and affordable cost of living^13^. The low cost, flexibility, ease of deployment wide and range of applications of the WSNs and IoD have all led to rise in smart city adoption^14^.

Although smart cities provide numerous services and merits, they are exposed to numerous security, performance and privacy challenges. For instance, a typical smart city is composed of numerous sensors and IoT devices that generate massive volumes of data. Some of these data items contain user-specific information such as habits, location and behavior. Since the collected data are exchanged over the public channels, they are susceptible to attacks^15–17^. In addition, some sensors and drones are placed in unattended environment but accessible locations and hence can be physically captured by the attackers^18^. Thereafter, the data stored in their memories can be extracted. Using the obtained credential, attackers can impersonate as legitimate entities. In addition, the authenticity of users, Cyber-Physical System (CPS), and Customer Premises Equipment (CPE) such as sensors and actuators is a major concern in smart cities. The high number of interconnected heterogeneous devices increases the surface from which adversaries can launch attacks, which can compromise economic development, safety and well-being of the users^19^. It is also possible for the collected data to be misused by the end users, posing serious threat to the smart cities^20^. Moreover, some of the devices in smart cities have vulnerabilities which can be exploited by the adversaries to steal data, gain unauthorized access and manipulate the systems.

Based on the above discussion, it is evident that security and privacy are key challenges that need to be solved in smart cities. There is therefore need for the development of robust security schemes that can protect privacy, authenticity and data integrity^17,21–24^. As explained in^25^, reliable data measurement is critical for most IoT applications. As such, there is need of ensuring that data is generated and transferred by only authorized users and devices. To this end, various authentication protocols have been developed for the smart cities. However, majority of them fail to offer user anonymity and are vulnerable to attacks such as Denial of Service (DoS)^13^. In addition, majority of these schemes deploy public key cryptography^26^ which is inefficient for the power and energy-limited smart city sensors. As such, the design of secure and truly lightweight security solutions for smart cities is still a challenging activity.

## Research contributions

- We leverage on biometrics, error correction codes and fuzzy commitment schemes to develop a secure and energy efficient authentication scheme for the smart cities.
- Unlike majority of the current schemes that deploy timestamps to prevent replay attacks, our protocol incorporates random nonces in all exchanged messages. This is demonstrated to address security issues such as de-synchronization attacks inherent in timestamp-based schemes.
- We execute extensive formal security analysis using the BAN logic to show that our scheme performs strong mutual authentication and key negotiation in an appropriate manner.
- Informal security analysis is carried out to demonstrate that the proposed protocol supports numerous functional and security features such as strong mutual authentication, anonymity and perfect key secrecy. In addition, this analysis shows that our scheme can withstand a myriad of smart city security threats such as session hijacking, privileged insider and side-channeling attacks.
- Elaborate comparative evaluations are carried out to show that the proposed protocol incurs the lowest computation overheads and hence is energy efficient.

The rest of this paper is structured as follows: “Related work” section discusses related works while “The proposed protocol” section presents the proposed protocol. On the other hand, “Security analysis” section discusses the security analysis of our scheme while “Performance evaluation” section describes its performance evaluation. Towards the end of this paper, “Conclusion and future work” section presents the conclusion and future research work.

### Mathematical preliminaries

In this section, we provide some mathematical formulations for the key cryptographic building blocks of the proposed scheme. This include fuzzy commitment, one way hashing and error correcting codes.

#### One way hashing

Suppose that ***N*** is a set of all positive integers, *P*_k_ is a family of uniform probability distributions and *ℒ* is a polynomial such that *ℒ* (*k*) > *k*. Then, *H* represents a family of functions which are defined by *H* = *P*_k_* H*_k_, where *H*_k_ is a multi-set of functions from ${\sum }^{\mathcal{L}(k)}$ to ${\sum }^{k}$. Here, *P*_k_ (x) = $1/{2}^{\mathcal{L}(k)}$ for all ${x\in \sum }^{\mathcal{L}(k)}$. H is referred to as a hash function, which compresses *ℒ* (*k*)-bit input into some *k*-bit output strings.

##### Definition 1

Let us consider two strings ${a,b\in \sum }^{\mathcal{L}(k)}$, where $a\ne b$. We say that string *a* collides with string *b* under $h\in {H}_{k}$, or (*a*, *b*) is a collision pair for *h*, provided that *h* (*a*) = *h* (*b*).

##### Definition 2

*H* is regarded as polynomial time computable on condition that there exists a polynomial (in *k*) time algorithm that derives all $h\in H.$

##### Definition 3

*H* is regarded as accessible provided that there exists a probabilistic time algorithm which takes input $k\in {\varvec{N}}$ and outputs homogeneously at random a depiction of $h\in {H}_{k}$.

#### Error correcting codes

In noisy transmission channels, error correcting code (*ecc*) is crucial for accurate reception of the transmitted data. Particularly, error correcting codes are critical in fuzzy commitment systems where they ensure that data is exchanged accurately over noisy transmission channels. Suppose that *Ψ* is a set of messages, where *Ψ* = {0,1}^φ^. Then, an error correcting code is made up of a set of codephrases $CP\subseteq \{\text{0,1}{\}}^{\rho }$. A typical *ecc* comprises of a translation function *ω* and decoding function *f*, where *ω*: *Ψ* → *CP* and *f*: {0,1}^ρ^ → *CP* $\cup$ {*γ*}. Denoting the Hamming distance as *ℌ*, then the decoding function maps a *ρ—*bit string *S* to the closest codephrase in *CP* in terms of *ℌ*, otherwise it outputs *γ*. Prior to transmission, any message *ψ* $\in \Psi$ is mapped to an element in *CP*. For improved redundancy, $\rho >\varphi$. Suppose that *θ* is the correction threshold, and *τ* $\in${0,1}^ρ^ is the error term. Then, for codephrase *cp* $\in$ *CP* and Hamming weight ||*τ*||≤ *θ*, we have *f* (*cp* ⊕ *τ*) = *cp*.

#### Fuzzy commitment

Due to the noisy nature of biometric data, the input biometrics is not exactly similar to the biometric templates. Therefore, the biometric template can be deployed in fuzzy commitment schemes. Suppose that *h*: {0,1}^ρ^ → {0,1}^χ^ is a collision-resistant one-way hashing function. We also let *w* be the witness, λ = *h*(*cp*) and *ε* = *w* ⊕ *cp*. Then, the fuzzy commitment scheme *F*: ({0,1}^ρ^, {0,1}^ρ^) → ({0,1}^χ^, {0,1}^ρ^) commits codephrase *cp* $\in$ *CP* using a *ρ –* bit witness *w* as *F* (*cp*,* w*) = (*λ*, *ε*). Provided that witness w^*^ is fairly close to *w* but not necessarily equivalent to *w*, then commitment *F* (*cp*,* w*) = (*λ*, *ε*) can be opened using w^*^. Suppose that this commitment is sent from *T* towards *R*. Therefore, the opening of this commitment at *R* using w^*^ involves the derivation of cp^*^ = *f* (w^*^ ⊕ *ε*). Since *ε* = *w* ⊕ *cp*, then cp^*^ can also be expressed as cp^*^ = *f* (*cp* ⊕ (w^*^ ⊕ *w*)). Thereafter,* R* confirms whether λ ≟ *h* (cp^*^). Provided that this condition holds, then the fuzzy commitment is effectively opened. Otherwise, witness w^*^ is flagged as invalid. We apply this fuzzy commitment concept in our biometric authentication procedures by treating the biometric template as witness *w*. As such, the user inputs biometric data (seen as witness w^*^) which is deployed to open codephrase *cp*, provided that w^*^ is closer to *w*.

### Attack model

In the proposed scheme, the adversary is assumed to have all the capabilities in the Canetti and Krawczyk (CK) threat model. Therefore, the communication process within the smart city is executed over the public internet and hence the attacker can have full control of this channel. In addition, the attacker can eavesdrop, alter, delete and insert bogus messages in the communication channel during message exchanges over the public smart city wireless channels. Moreover, all the sensitive data stored in the sensor nodes can be extracted upon physical capture of these nodes. It is also possible for all secret information, ephemeral secrets and session states to be compromised via session-hijacking attacks.

## Related work

Many security techniques have been developed over the recent past to offer security protection in IoT and other devices interconnected in smart cities^27–31^. However, these schemes have extensive communication and computation overheads^32^. Although the protocol in^33^ is lightweight and hence can address this issue, it cannot withstand outsider attackers^34^. Blockchain technology^35^ can provide authentication and decentralized management of identity as well as authorization policies. Therefore, many blockchain-based security schemes have been presented in^36–43^. However, these schemes incur high storage and computation overheads which are not suitable for the sensors^44^. Therefore, a lightweight authentication scheme is developed in^3^. However, the communication costs analysis of this scheme is missing. In addition, it has not been evaluated against attacks such as side-channeling and de-synchronization.

Based on the Physically Unclonable Function (PUF), mutual authentication schemes are presented in^4,45,46^. Although these protocols can withstand physical capture and side-channeling attacks, PUF-based schemes have stability challenges^47^. On the other hand, biometric-based schemes have been introduced in^48–51^. However, the three-factor authentication protocol in^48^ cannot preserve perfect backward secrecy^52^. Therefore, an improved scheme is presented in^52^. Unfortunately, this protocol is susceptible to offline password guessing, forgery, session key disclosure and replay attacks^49^. In addition, it cannot uphold perfect forward secrecy and data confidentiality. On the other hand, the protocol in^50^ is vulnerable to impersonation and stolen verifier attacks^51^. In addition, it fails to preserve user untraceability. To prevent single-point of failure attacks, a scheme that is devoid of trusted issuer is developed in^53^. However, comparative security and performance analyses of this scheme have not been carried out. Similarly, feasibility, scalability and comparative analyses against the state of the art techniques are missing in^54^.

To mitigate service-oriented attacks in smart cities, a context-based trust model is presented in^55^. However, processing huge volumes of contextual data results in high computation overhead^56^. Similarly, the quantum-inspired technique presented in^57^ incurs extensive computation overheads due to the required quantum computing^58^. Although an energy-efficient framework for IoT developed in^59^ can address this issue, its comparative performance and security analyses have not be carried out. The verification scheme in^60^ is efficient and hence can address the performance issues in^55,57^. However, it fails to provide robust identity check and user anonymity^61^. Similarly, the Elliptic Curve Cryptography (ECC) based protocol in^61^ cannot offer anonymity and untraceability. Therefore, an ECC based anonymous authentication protocol is introduced in^13^, while an identity based technique is presented in^62^ to offer strong unforgeability and anonymity. Although the scheme in^13^ is shown to resist DoS attacks, its numerous point multiplications can lead to high computation costs. Similarly, the fuzzy extractor based protocol in^63^ incurs heavy computation overheads^32^. On the other hand, identity-based schemes have key escrow problems^64^.

To protect smart cities against botnet attacks, an algorithm based on Long Short-Term Memory (LSTM) is developed in^65^. However, its evaluation is carried out on a single dataset of botnet attacks and hence fails to reflect a variety of attack vectors in a typical smart city. In addition, its performance evaluation in terms of the required resources has not been presented. To ensure access control and high security level, Public Key Cryptography (PKC) based protocols have been developed in^66–68^. However, these schemes are susceptible to physical capture attacks and hence their stored secret credentials can be retrieved^4^. Thereafter, the attackers are able to impersonate the entities whose credentials have been extracted. In addition, most of these PKC-based schemes incur extensive communication and computation overheads^69^. Moreover, the homomorphic encryption based protocol in^66^ is vulnerable to privileged insider and session key disclosure attacks^4^. On its part, the bilinear pairing based protocol in^67^ fails to offer perfect forward secrecy and cannot withstand impersonation attacks^68^. In addition, the deployed bilinear pairing operations incur extensive communication and computation overheads and hence cannot support real-time services provision in smart cities. Regarding the ECC-based developed in^68^, it is susceptible to impersonation, replay and privileged insider attacks^70^. In addition, it cannot offer strong mutual authentication among the communicating entities. Therefore, an improved security technique is presented in^70^. However, this protocol is vulnerable to attacks such as server spoofing, session key disclosure and forgery^4^. Although the schemes in^71,72^ can solve some of these challenges, they have not been evaluated against de-synchronization attacks. On their part, the three-factor security schemes in^48–52^ are susceptible to potential security attacks^4^. Although the protocol in^73^ addresses some of the attacks such as ephemeral leakage, it cannot withstand identity guessing attacks^74–76^.

Based on the discussion above, it is evident that many schemes have been developed for the smart city environment. However, the attainment of perfect smart city security at low computation and communication is still an open challenge. For instance, many security protocols have been shown to be vulnerable to numerous attacks while others cannot support anonymity, mutual authentication and untraceability. In addition, some of these schemes do not incorporate biometric and password change procedures. Moreover, some of these security techniques incur extensive computation and communication overheads while others deploy centralized architecture which can easily result in central failure, denial of services and privacy breaches^39^. The proposed protocol is demonstrated to address some of these security, performance and privacy challenges. For instance, our scheme incurs the lowest computation overheads among its peers and hence addresses performance challenges in most of the above protocols. In addition, it provides support for anonymity, mutual authentication and untraceability which are features missing in most of the above schemes. Moreover, it mitigates attacks which are rarely considered in most of the existing protocols. Such attacks include de-synchronization, eavesdropping, session hijacking, forgery and side-channeling.

## The proposed protocol

The elliptic curve cryptography offer offers strong security at relatively shorter key sizes compared to other public key cryptographies such as RSA. Therefore, we deploy elliptic curve cryptography in the proposed scheme. To address physical and side-channeling attacks, we leverage on biometric, error correction codes and fuzzy commitment schemes.

### Motivation

Smart cities have streamlined services in urban centers, leading to the enhancement on the quality of life of the citizens. In a typical smart city, numerous smart devices are interconnected to facilitate activities such as surveillance, shipping, logistics, healthcare and warehousing. As such, high volumes of data are generated and exchanged among these smart devices. Since these message exchanges are carried out over the public internet, many security and privacy threats lurk in this environment. For instance, personal user information can be eavesdropped over the public channels while successful sensor and device capture can facilitate impersonation attacks. Therefore, past research works have presented numerous security techniques to alleviate these challenges. Unfortunately, majority of these schemes are based on computationally extensive cryptographic operations such as bilinear pairings. Consequently, these schemes are inefficient for the computation, bandwidth, storage and energy constrained sensor nodes. In addition, some of the presented security solutions still have security and privacy related issues^77,78^ such as susceptibility to physical, impersonation, privileged insider and Man-in-the-Middle (MitM) attacks. Therefore, the design of provably secure and yet efficient^79^ authentication protocols for smart cities is a nontrivial challenge.

### Requirements

In smart city environment, security efficiency^80^ is critical in ensuring that users can authenticate and access the required data in a timely manner. This is particularly important due to the bandwidth, energy, computation power and storage constraints of the interconnected sensor networks in light of this, the proposed protocol must fulfill the following security and performance requirements.

*Mutual authentication* All the entities involved in message exchanges within the smart city must verify each other at the onset of the communication process.

*Key agreement* Upon successful validation of each other, session keys should be setup among the communicating parties. This key is deployed to encipher all the exchanged data within the smart city.

*Perfect key secrecy* It should be computationally infeasible for the adversary to capture the current session keys and utilize them to derive keys for the previous and subsequent sessions.

*Anonymity* The adversaries with the capabilities of eavesdropping the communication channel should not be in a position to obtain the real identities of the communicating parties.

*Untraceability* An adversary should be unable to associate any communication sessions to a particular network entity.

*Resilience against threats* typical security threats such as de-synchronization, denial of service, physical, eavesdropping, session hijacking, privileged insider, KSSTI, replays, forgery, MitM, impersonation and side-channeling should be curbed in our scheme.

*Resource efficiency* Owing to the resource-constrained nature of the smart city sensors and devices, the proposed scheme should be computationally efficient.

**Figure 1:**
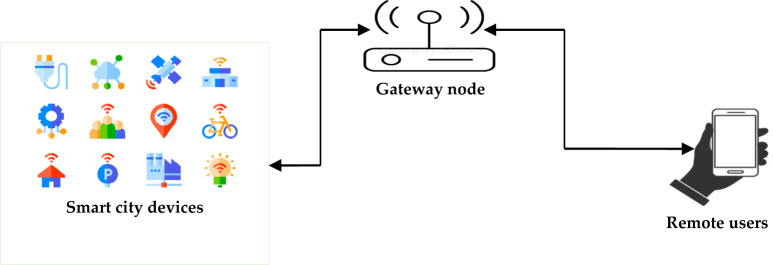
Smart city network model.

In our scheme, each user deploys his/her mobile device (*MD*_i_) to interact with the smart city sensor *SN*_j_ through some gateway node *GW*_k_. In this environment, the *GW*_k_ bridges the connection between *MD*_i_ and *SN*_j_ as shown in Fig. 1.

Table 1 presents all the notations deployed throughout this paper. The major phases executed in our scheme include the system setup, registration, login, authentication, key negotiation, and password change. The sub-sections below describe these phases in greater details.

**Table 1:** Notations.

Symbol	Description
*GW*_k_	Gateway node *k*
*SN*_j_	Sensor node *j*
*MD*_i_	User’s mobile device *i*
*n*	Secret key for *GW*_k_
*P*_k_	Public key for *GW*_k_
*M*_k_	Master key for *GW*_k_
*SNID*_j_	Unique identity for sensor node *j*
*K*_GS_	Secret key shared between *GW*_k_ and *SN*_j_
*U*_i_	User *i*
*UID*_i_	Unique identity for user *i*
*PW*_i_	Password for user *i*
*SK*_S_	Session key derived at *SN*_j_
*SK*_G_	Session key derived at the *GW*_k_
*SK*_D_	Session key derived at the *MD*_i_
*h* (.)	One-way hashing function
||	Concatenation operation
⊕	XOR operation

### System setup

This phase is carried out by the gateway node *GW*_k_. The goal is to derive the long term keys that will be utilized in the latter phases of our scheme. The following 3 steps are executed during the system setup phase.

*Step 1* The *GW*_k_ selects some elliptic curve *E* and additive group *G* over finite field *F*_p_. Here, the generator is point *P* whose order is a large prime number *q*.

*Step 2* *GW*_k_ generates nonce *n* $\in {Z}_{q}^{*}$ and sets it as its secret key. Next, it derives its corresponding public key as *P*_k_ = *nP*.

*Step 3* The *GW*_k_ selects *M*_k_ as its master key and privately keeps both* n* and *M*_k_. Finally, it publishes parameter set {*P*,* P*_k_, *G*, *E* (*F*_p_)}.

### Sensor node registration

Prior to actual deployment in their application domains, each sensor node *SN*_j_ must be registered at the gateway node *GW*_k_. The aim is to assign these sensors some security values that are deployed during the login, authentication and key negotiation phase. The following 2 steps are executed in this phase.

**Figure 2:**
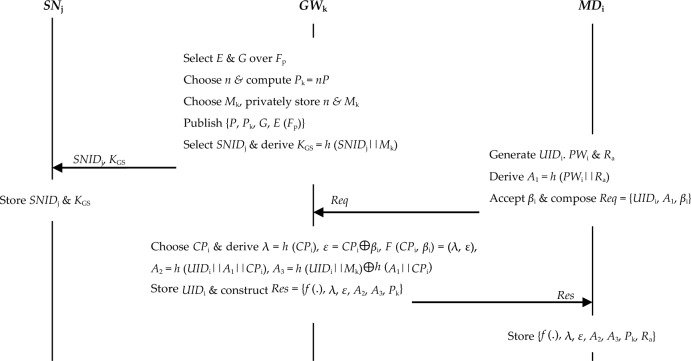
System setup and registration.

*Step 1* The *GW*_k_ chooses *SNID*_j_ as sensor node *SN*_j_ unique identity. This is followed by the derivation of private key *K*_GS_ = *h* (*SNID*_j_||*M*_k_). *GW*_k_ sends values *SNID*_j_ and *K*_GS_ to *SN*_j_ over secure channels as shown in Fig. 2.

*Step 2* Upon receiving parameters *SNID*_j_ and *K*_GS_ from the *GW*_k_, the *SN*_j_ stores them in its memory. The sensor node is now ready to be deployed to the field.

### User registration

All users within the smart city network must be registered at their respective gateway nodes. During this phase, the users are assigned security tokens that they will deploy to securely acquire data from the sensor devices deployed in a given domain. The following 4 steps are executed during this process.

*Step 1* The user *U*_i_ through the *MD*_i_ generates unique identity *UID*_i_ and password *PW*_i_. Next, nonce *R*_a_ is generated which is then used to derive value *A*_1_ = *h* (*PW*_i_||*R*_a_).

*Step 2* The *U*_i_ imprints biometric data *β*_i_ onto the *MD*_i_. Finally, registration request *Req* = {*UID*_i_,* A*_1_,* β*_i_} is constructed and forwarded to the *GW*_k_ over secure channels as shown in Fig. 2.

*Step 3* Upon receiving registration request *Req* from *U*_i_, the *GW*_k_ selects some random codephrase *CP*_i_ $\in$ *CP* for this particular user* U*_i_. Next, it derives tokens λ = *h* (*CP*_i_), *ε* = *CP*_i_ ⊕ *β*_i_, *F* (*CP*_i_,* β*_i_) = (λ, ε), *A*_2_ = *h* (*UID*_i_||*A*_1_||*CP*_i_) and *A*_3_ = *h* (*UID*_i_||*M*_k_) ⊕ *h* (*A*_1_||*CP*_i_). Finally, it stores *UID*_i_ in its database before composing registration response *Res* = {*f* (.), λ,* ε*,* A*_2_,* A*_3_,* P*_k_} that is sent to the* U*_i_ over secured channels.

*Step 4* After getting registration response *Res* from the *GW*_k_, the* U*_i_ through *MD*_i_ stores value set {*f* (.), λ,* ε*,* A*_2_,* A*_3_,* P*_k_,* R*_a_} in its memory.

### Login, authentication and key negotiation

This phase is activated whenever the user *U*_i_ through the *MD*_i_ wants some access to the data help by the sensors. Here, the security tokens assigned during the registration phase are deployed to authenticate* U*_i_ to the gateway node *GW*_k_. To accomplish this, the following 8 steps are executed.

*Step 1* User *U*_i_ imprints his/her biometric data *β*_i_^*^ onto the *MD*_i_ upon which value *CP*_i_^*^ = *f* (*ε*⊕* β*_i_^*^) is computed. Since *ε* = *CP*_i_⊕*β*_i_, *CP*_i_^*^ can also be expressed as *CP*_i_^*^ = *f*(*CP*_i_⊕(*β*_i_⊕*β*_i_^*^)). Thereafter, the *MD*_i_ checks whether *h* (*CP*_i_^*^) ≟ λ = *h* (*CP*_i_). Basically, the user login session is terminated upon verification failure. Otherwise, *U*_i_ has passed the biometric validation and hence proceeds to input unique identity *UID*_i_ and password *PW*_i_ into the *MD*_i_.

*Step 2* The *MD*_i_ computes *A*_2_^*^ = *h* (*UID*_i_||*h* (*PW*_i_||*R*_a_)||*CP*_i_^*^) and confirms whether *A*_2_^*^≟* A*_2_. Since *A*_1_ = *h* (*PW*_i_||*R*_a_), this verification should be successful otherwise the session is aborted. However, if this validation is successful, both user identity and password have been authenticated by the *MD*_i_.

**Figure 3:**
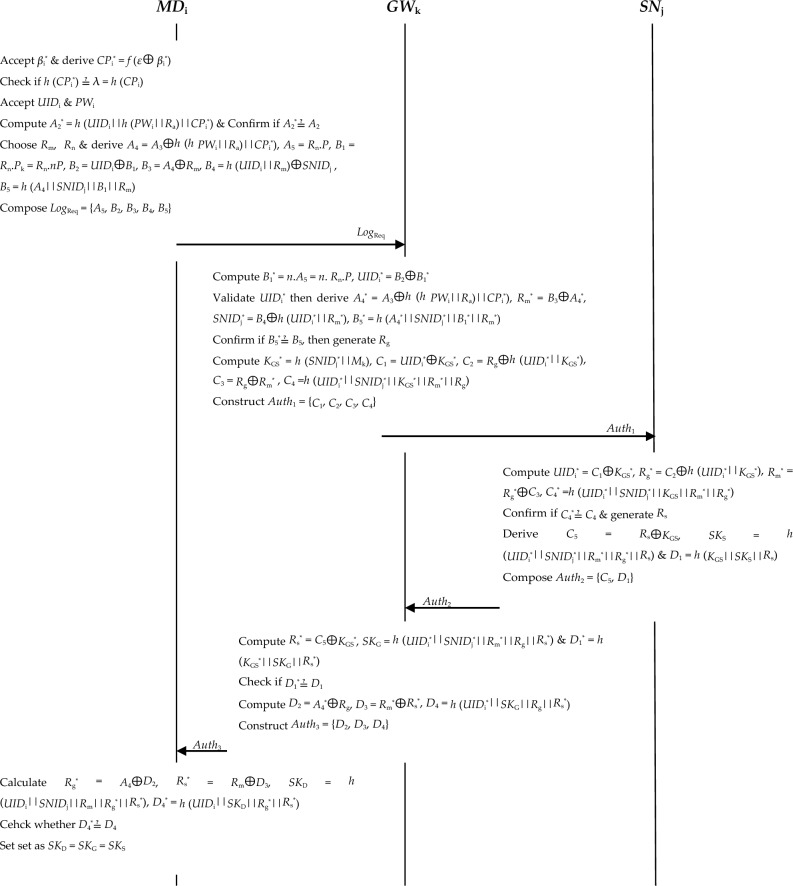
Login, authentication and key negotiation.

*Step 3* The *MD*_i_ selects nonce *R*_m_ and *R*_n_ $\in {Z}_{q}^{*}$ and computes values *A*_4_ = *A*_3_ ⊕ *h* (*h PW*_i_||*R*_a_)||*CP*_i_^*^), *A*_5_ = *R*_n_.*P*, *B*_1_ = *R*_n_.*P*_k_ = *R*_n_.*nP*, *B*_2_ = *UID*_i_ ⊕ *B*_1_, *B*_3_ = *A*_4_ ⊕ *R*_m_, *B*_4_ = *h* (*UID*_i_||*R*_m_) ⊕ *SNID*_j_ and *B*_5_ = *h* (*A*_4_||*SNID*_j_||*B*_1_||*R*_m_). At the end, the *MD*_i_ constructs login request message *Log*_Req_ = {*A*_5_,* B*_2_,* B*_3_,* B*_4_,* B*_5_} that is transmitted to the *GW*_k_ over public channels as shown in Fig. 3.

*Step 4* Upon receiving login request message *Log*_Req_, the *GW*_k_ derives values *B*_1_^*^ = *n*.*A*_5_ = *n*.* R*_n_.*P*, *UID*_i_^*^ = *B*_2_⊕*B*_1_^*^. This is followed by the confirmation of whether *UID*_i_^*^ is in its database. Provided that *UID*_i_^*^ cannot be found in its database, the *MD*_i_ login request is rejected. Otherwise, the *GW*_k_ calculates *A*_4_^*^ = *A*_3_⊕*h* (*h PW*_i_||*R*_a_)||*CP*_i_^*^), *R*_m_^*^ = *B*_3_⊕*A*_4_^*^, *SNID*_j_^*^ = *B*_4_⊕*h* (*UID*_i_^*^||*R*_m_^*^) and *B*_5_^*^ = *h* (*A*_4_^*^||*SNID*_j_^*^||*B*_1_^*^||*R*_m_^*^).

*Step 5* The *GW*_k_ checks if *B*_5_^*^≟* B*_5_ such that the session is terminated if this condition does not hold. Otherwise, it generates nonce *R*_g_ and derives values *K*_GS_^*^ = *h* (*SNID*_j_^*^||*M*_k_), *C*_1_ = *UID*_i_^*^ ⊕ *K*_GS_^*^, *C*_2_ = *R*_g_ ⊕ *h* (*UID*_i_^*^||*K*_GS_^*^), *C*_3_ = *R*_g_ ⊕ *R*_m_^*^ and *C*_4_ = *h* (*UID*_i_^*^||*SNID*_j_^*^||*K*_GS_^*^||*R*_m_^*^||*R*_g_). At last, it composes authentication message *Auth*_1_ = {*C*_1_,* C*_2_,* C*_3_,* C*_4_} which is sent to the sensor node *SN*_j_ over public channels.

*Step 6* On receiving authentication message *Auth*_1_, the *SN*_j_ derives *UID*_i_^*^ = *C*_1_ ⊕ *K*_GS_^*^,* R*_g_^*^ = *C*_2_ ⊕ *h* (*UID*_i_^*^||*K*_GS_^*^), *R*_m_^*^ = *R*_g_^*^ ⊕ *C*_3_ and *C*_4_^*^ = *h* (*UID*_i_^*^||*SNID*_j_^*^||*K*_GS_||*R*_m_^*^||*R*_g_^*^). Next, it checks if *C*_4_^*^≟* C*_4_ such that the session is aborted upon verification failure. Otherwise, the *SN*_j_ generates nonce *R*_s_ before calculating parameter *C*_5_ = *R*_s_ ⊕ *K*_GS_, session key *SK*_S_ = *h* (*UID*_i_^*^||*SNID*_j_^*^||*R*_m_^*^||*R*_g_^*^||*R*_s_) and value *D*_1_ = *h* (*K*_GS_||*SK*_S_||*R*_s_). Finally, *SN*_j_ constructs authentication response message *Auth*_2_ = {*C*_5_,* D*_1_} which is sent over to *GW*_k_.

*Step 7* After getting authentication response message *Auth*_2_, the *GW*_k_ derives value* R*_s_^*^ = *C*_5_ ⊕ *K*_GS_^*^, session key *SK*_G_ = *h* (*UID*_i_^*^||*SNID*_j_^*^||*R*_m_^*^||*R*_g_||*R*_s_^*^) and parameter *D*_1_^*^ = *h* (*K*_GS_^*^||*SK*_G_||*R*_s_^*^). This is followed by the confirmation of whether* D*_1_^*^≟* D*_1_ such that the session is terminated upon verification failure. Otherwise, the *GW*_k_ derives parameters *D*_2_ = *A*_4_^*^ ⊕ *R*_g_, *D*_3_ = *R*_m_^*^ ⊕ *R*_s_^*^ and *D*_4_ = *h* (*UID*_i_^*^||*SK*_G_||*R*_g_||*R*_s_^*^). At last, it composes authentication message *Auth*_3_ = {*D*_2_, *D*_3_, *D*_4_} that is forwarded to the *MD*_i_.

*Step 8* On receiving authentication message *Auth*_3_, the *MD*_i_ calculates *R*_g_^*^ = *A*_4_ ⊕ *D*_2_, *R*_s_^*^ = *R*_m_ ⊕ *D*_3_, session key *SK*_D_ = *h* (*UID*_i_||*SNID*_j_||*R*_m_||*R*_g_^*^||*R*_s_^*^) and value *D*_4_^*^ = *h* (*UID*_i_||*SK*_D_||*R*_g_^*^||*R*_s_^*^). It then verifies whether *D*_4_^*^≟* D*_4_ such that the session is aborted upon validation failure. Otherwise, user *U*_i_, *GW*_k_ and *SN*_j_ have successfully authenticated each other and negotiated session keys. As such, the session key is set as *SK*_D_ = *SK*_G_ = *SK*_S_ and is shared among these three entities. Afterwards, *U*_i_ can securely access sensed data held at *SN*_j_ vial *GW*_k_.

### Password change

In this phase, the user executes password change upon its compromise. To reduce on communication overheads, this change is carried out without contacting the gateway node *GW*_k_. the following…steps are executed during this phase.

*Step 1* The user *U*_i_ imprints biometric data *β*_i_^*^onto the *MD*_i_. Thereafter, the *MD*_i_ derives *CP*_i_^*^ = *f* (*ε* ⊕ *β*_i_^*^) = *f*(*CP*_i_ ⊕ (*β*_i_ ⊕ *β*_i_^*^)).Next, the *MD*_i_ validates whether *h* (*CP*_i_^*^) ≟ λ = *h* (*CP*_i_) such that the password change session is terminated upon verification failure. Otherwise, the user *U*_i_ has passed biometric authentication.

*Step 2* User *U*_i_ inputs *UID*_i_ and *PW*_i_ into the *MD*_i_ after which it calculates *A*_2_^*^ = *h* (*UID*_i_||*h* (*PW*_i_||*R*_a_)||*CP*_i_^*^). This is followed by the confirmation of whether *A*_2_^*^≟* A*_2_ such that the session is aborted upon verification failure. Otherwise, user *U*_i_ is prompted to input new password *PW*_i_^New^.

*Step 3* The *MD*_i_ computes *A*_2_^New^ = *h* (*UID*_i_||*h* (*PW*_i_^New^||*R*_a_)||*CP*_i_^*^) and *A*_3_^New^ = *A*_3_ ⊕ *h* (*h* (*PW*_i_||*R*_a_)||*CP*_i_^*^) ⊕ *h* (*h* (*PW*_i_^New^||*R*_a_)||*CP*_i_^*^). Finally, the *MD*_i_ updates value set {*A*_2_,* A*_3_} with their refreshed counterparts {*A*_2_^New^,* A*_3_^New^} in its memory.

## Security analysis

In this section, we formally and informally analyze the security features provided by the proposed scheme. Whereas the formal security analysis is executed using Burrows–Abadi–Needham logic (BAN) logic, informal security analysis is carried out by formulating and proofing some propositions.

### Formal security analysis

The aim of this sub-section is to verify that our scheme performs strong mutual authentication and key negotiation in an appropriate manner. The notations used throughout this proof are described below.

# (A): *A* is fresh.

${\langle \text{A}\rangle }_{\text{B}}$ : *A* is enciphered using *B.*

S|≡Y: *S* believes *Y.*

(A, B): *A* or *B* is part of message (*A*, *B*).

S ◁ Y: *S* sees *Y*.

S|~ A: *S* once said *A*.

(A, B)_µ_:* A* or *B* is hashed using *µ*.

S $\Rightarrow$ A: *S* has jurisdiction over *A*.

$\text{S}\stackrel{ \mu }{\leftrightarrow }\text{T}$ : *S* and *T* communicate using shared key *µ*.

In addition to the above BAN logic rules, the following BAN logic rules are used in our proof.

*Belief Rule (BR):* $\frac{S|\equiv \left(A\right),S|\equiv \left(B\right)}{S|\equiv (A, B)}$

*Message Meaning Rule (MMR):* $\frac{{S| \equiv {\text{S}}\mathop \leftrightarrow \limits^{\mu } {\text{T}},{\text{S}} \triangleleft \langle {\text{A}}\rangle _{{\mu }} }}{{S| \equiv T|\sim A}}$

*Session Keys Rule (SKR):* $\frac{S|\equiv \#\left(A\right),S|\equiv T|\equiv A}{S|\equiv \text{S}\stackrel{ \mu }{\leftrightarrow }\text{T}}$

*Jurisdiction Rule (JR):* $\frac{S|\equiv T\Rightarrow A,S|\equiv T|\equiv A}{S|\equiv A}$

*Fresh Promotion Rule (FPR):* $\frac{S|\equiv \#(A)}{S|\equiv \#(A,B)}$

*Nonce Verification Rule (NVR):* $\frac{S|\equiv \#\left(A\right),S|\equiv T|\sim A}{S|\equiv T|\equiv A}$

To be secure under the BAN logic, the proposed scheme must satisfy the following security goals.

Goal 1: *SN*_j_ $|\equiv$ *SN*_j_ $\stackrel{ {SK}_{S}}{\leftrightarrow }$* MD*_i_

Goal 2: *SN*_j_ $|\equiv$ *MD*_i_ $|\equiv$ *SN*_j_ $\stackrel{ {SK}_{S}}{\leftrightarrow }$* MD*_i_

Goal 3: *MD*_i_ $|\equiv$ *SN*_j_ $\stackrel{ {SK}_{D}}{\leftrightarrow }$* MD*_i_

Goal 4: *MD*_i_ $|\equiv$ *SN*_j_ $|\equiv$ *SN*_j_ $\stackrel{ {SK}_{D}}{\leftrightarrow }$* MD*_i_

Goal 5: *GW*_k_ $|\equiv$ *GW*_k_ $\stackrel{ {SK}_{G}}{\leftrightarrow }$ *MD*_i_

Goal 6: *GW*_k_ $|\equiv$ *MD*_i_ $|\equiv$ *GW*_k_ $\stackrel{ {SK}_{G}}{\leftrightarrow }$ *MD*_i_

Goal 7: *GW*_k_ $|\equiv$ *GW*_k_ $\stackrel{ {SK}_{G}}{\leftrightarrow }$ *SN*_j_

Goal 8: *GW*_k_ $|\equiv$ *SN*_j_ $|\equiv$ *GW*_k_ $\stackrel{ {SK}_{G}}{\leftrightarrow }$ *SN*_j_

In our scheme, 4 messages are exchanged during the login, authentication and key agreement phase. These messages include *Log*_Req_ = {*A*_5_,* B*_2_,* B*_3_,* B*_4_,* B*_5_}, *Auth*_1_ = {*C*_1_,* C*_2_,* C*_3_,* C*_4_}, *Auth*_2_ = {*C*_5_,* D*_1_} and *Auth*_3_ = {*D*_2_, *D*_3_, *D*_4_}. For ease of analysis, we transform these messages into idealized format as follows.

*MD*_i_ → *GW*_k_: *Log*_Req_ = {*A*_5_,* B*_2_,* B*_3_,* B*_4_,* B*_5_}

*Idealized format*: {*R*_n_.*P*, $${\langle {UID}_{i}\rangle }_{{R}_{n}.{P}_{k}},{{\langle {R}_{m}\rangle }_{h({UID}_{i}||{M}_{k})},\langle {SNID}_{j}\rangle }_{h({UID}_{i}|\left|{R}_{\text{m}}\right)},({SNID}_{j}||{R}_{\text{m}}{)}_{{R}_{n}.{P}_{k}}{,}_{h({UID}_{i}||{M}_{k})}$$}

*GW*_k_ → *SN*_j_: *Auth*_1_ = {*C*_1_,* C*_2_,* C*_3_,* C*_4_}

*Idealized format*: {$${\langle {UID}_{i}^{*}\rangle }_{{KG}_{S}}, {\langle {R}_{g}\rangle }_{h({UID}_{i}^{*}|\left|{KG}_{S}\right)},{\langle {R}_{m}\rangle }_{{R}_{g}}, ({UID}_{i}||{SNID}_{j}{)}_{({R}_{m},{ R}_{g},{ KG}_{S})}$$}

*SN*_j_ → *GW*_k_: *Auth*_2_ = {*C*_5_,* D*_1_}

*Idealized format*: {$${\langle {R}_{s}\rangle }_{{KG}_{S}}$$, ($${R}_{s}{)}_{({SK}_{S},{ KG}_{S})}$$

*GW*_k_ → *MD*_i_: *Auth*_3_ = {*D*_2_, *D*_3_, *D*_4_}

*Idealized format*: {$${\langle {R}_{g}\rangle }_{h({UID}_{i}|\left|{KG}_{S}\right)}$$,$${\langle {R}_{s}^{*}\rangle }_{{R}_{m}^{*}}$$, ($${UID}_{i}^{*}{)}_{({R}_{g},{ R}_{s}^{*}, { SK}_{G})}$$}

The following initial state assumptions (SA) are also made.

SA_1_: *U*_i_ $$|\equiv$$# *R*_m_

SA_2_: *GW*_k_ $$|\equiv$$# *R*_g_

SA_3_: *SN*_j_ $$|\equiv$$# *R*_s_

SA_4_: *MD*_i_ $|\equiv$ *MD*_i_ $\stackrel{ {nR}_{n}.P}{\leftrightarrow }$ *GW*_k_

SA_5_: *MD*_i_ $|\equiv$ *MD*_i_ $\stackrel{ {SK}_{S }}{\leftrightarrow }$ *SN*_j_

SA_6_: *GW*_k_ $|\equiv$* GW*_k_ $\stackrel{ {R}_{n}.nP}{\leftrightarrow }$ *MD*_i_

SA_7_: *GW*_k_ $|\equiv$* GW*_k_ $\stackrel{ {KG}_{S}}{\leftrightarrow }$ *SN*_j_

SA_8_: *SN*_j_ $|\equiv$* SN*_j_ $\stackrel{ {SK}_{S }}{\leftrightarrow }$ *MD*_i_

SA_9_: *SN*_j_ $|\equiv$* SN*_j_ $\stackrel{ {KG}_{S}}{\leftrightarrow }$ *GW*_k_

SA_10_: *MD*_i_ $|\equiv$* SN*_j_ $\Rightarrow$* R*_s_, *SK*_S_

SA_11_: *MD*_i_ $|\equiv$* GW*_k_ $\Rightarrow$* R*_g_, *SK*_G_

SA_12_: *GW*_k_ $|\equiv$* MD*_i_ $\Rightarrow$* R*_m_, *SK*_D_,*nR*_n_*P*

SA_13_: *GW*_k_ $|\equiv$* SN*_j_ $\Rightarrow$* R*_s_ ⊕ *KG*_S_

SA_14_: *SN*_j_ $|\equiv$* GW*_k_ $\Rightarrow$* R*_g_ ⊕ *h*(*UID*_i_||*KG*_S_)

SA_15_: *SN*_j_ $|\equiv$* MD*_i_ $\Rightarrow$* R*_m_, *SK*_D_

Based on the above BAN logic rules, idealized format of the exchanged messages and the initial state assumptions, we proof that the proposed scheme attains all the above security goals through the following BAN logic proof (*BLP*).

Using the idealized form of *Log*_Req_ and *BR*, we obtain *BLP*_1_,

*BLP*_1_: *GW*_k_ ◁ {*R*_n_.*P*, $${\langle {UID}_{i}\rangle }_{{R}_{n}.{P}_{k}},{{\langle {R}_{m}\rangle }_{h({UID}_{i}||{M}_{k})},\langle {SNID}_{j}\rangle }_{h({UID}_{i}|\left|{R}_{\text{m}}\right)},({SNID}_{j}||{R}_{\text{m}}{)}_{{R}_{n}.{P}_{k}}{,}_{h({UID}_{i}||{M}_{k})}$$}

Based on *SA*_6_, *BLP*_1_ and *MMR*, we obtain *BLP*_2_ as follows,

*BLP*_2_: *GW*_k_ $|\equiv$* MD*_i_ ~ {*R*_n_.*P*, ${\langle {UID}_{i}\rangle }_{{R}_{n}.{P}_{k}},{{\langle {R}_{m}\rangle }_{h({UID}_{i}||{M}_{k})},\langle {SNID}_{j}\rangle }_{h({UID}_{i}|\left|{R}_{\text{m}}\right)},({SNID}_{j}||{R}_{\text{m}}{)}_{{R}_{n}.{P}_{k}}{,}_{h({UID}_{i}||{M}_{k})}$}

Using *FPR* and *NVR* on both *BLP*_2_ and *SA*_1_ yields *BLP*_3_ as shown below.

*BLP*_3_: *GW*_k_ $|\equiv$* MD*_i_ $|\equiv$ {*R*_n_.*P*, ${\langle {UID}_{i}\rangle }_{{R}_{n}.{P}_{k}},{{\langle {R}_{m}\rangle }_{h({UID}_{i}||{M}_{k})},\langle {SNID}_{j}\rangle }_{h({UID}_{i}|\left|{R}_{\text{m}}\right)},({SNID}_{j}||{R}_{\text{m}}{)}_{{R}_{n}.{P}_{k}}{,}_{h({UID}_{i}||{M}_{k})}$}

On the other hand, using *JR* on *BLP*_3_, *SA*_6_ and *SA*_12_ yields *BLP*_4_.

*BLP*_4_: *GW*_k_ $$|\equiv$$ {*R*_n_.*P*, $${\langle {UID}_{i}\rangle }_{{R}_{n}.{P}_{k}},{{\langle {R}_{m}\rangle }_{h({UID}_{i}||{M}_{k})},\langle {SNID}_{j}\rangle }_{h({UID}_{i}|\left|{R}_{\text{m}}\right)},({SNID}_{j}||{R}_{\text{m}}{)}_{{R}_{n}.{P}_{k}}{,}_{h({UID}_{i}||{M}_{k})}$$}

Based on *BLP*_4_, the *SKR* is applied to obtain *BLP*_5_.

*BLP*_5_: *GW*_k_ $|\equiv$ *GW*_k_ $\stackrel{ {SK}_{G}}{\leftrightarrow }$ *MD*_i_, hence security **Goal 5** is attained.

On the other hand, *NVR* is applied to both *BLP*_5_ and *SA*_12_ to yield *BLP*_6_.

*BLP*_6_: *GW*_k_ $|\equiv$ *MD*_i_ $|\equiv$ *GW*_k_ $\stackrel{ {SK}_{G}}{\leftrightarrow }$ *MD*_i_, achieving security **Goal 6**.

Considering idealized formats of both *Auth*_1_ and *Auth*_3_, the application of *BR* yields *BLP*_7_ and *BLP*_8_.

*BLP*_7_: *SN*_j_ $$\triangleleft$${$${\langle {UID}_{i}^{*}\rangle }_{{KG}_{S}}, {\langle {R}_{g}\rangle }_{h({UID}_{i}^{*}|\left|{KG}_{S}\right)},{\langle {R}_{m}\rangle }_{{R}_{g}}, ({UID}_{i}||{SNID}_{j}{)}_{({R}_{m},{ R}_{g},{ KG}_{S})}$$}

*BLP*_8_: *MD*_i_ $$\triangleleft$${$${\langle {R}_{g}\rangle }_{h({UID}_{i}|\left|{KG}_{S}\right)}$$,$${\langle {R}_{s}^{*}\rangle }_{{R}_{m}^{*}}$$, ($${UID}_{i}^{*}{)}_{({R}_{g},{ R}_{s}^{*}, { SK}_{G})}$$}

Using the *MMR* on both *BLP*_7_ and *SA*_9_ results in *BLP*_9_.

*BLP*_9_: *SN*_j_ $|\equiv$ *GW*_k_ ~ {${\langle {UID}_{i}^{*}\rangle }_{{KG}_{S}}, {\langle {R}_{g}\rangle }_{h({UID}_{i}^{*}|\left|{KG}_{S}\right)},{\langle {R}_{m}\rangle }_{{R}_{g}}, ({UID}_{i}||{SNID}_{j}{)}_{({R}_{m},{ R}_{g},{ KG}_{S})}$}

However, the application of *MMR* on both *BLP*_8_ and *SA*_4_ yields *BLP*_10_.

*BLP*_10_: *MD*_i_ $$|\equiv$$ *GW*_k_ ~ {$${\langle {R}_{g}\rangle }_{h({UID}_{i}|\left|{KG}_{S}\right)}$$,$${\langle {R}_{s}^{*}\rangle }_{{R}_{m}^{*}}$$, ($${UID}_{i}^{*}{)}_{({R}_{g},{ R}_{s}^{*}, { SK}_{G})}$$}

Based on *BLP*_9_, *SA*_2_, *SA*_14_, *FPR* and the *NVR*, we obtain *BLP*_11_.

*BLP*_11_: *SN*_j_ $|\equiv$ *GW*_k_ $|\equiv$ {${\langle {UID}_{i}^{*}\rangle }_{{KG}_{S}}, {\langle {R}_{g}\rangle }_{h({UID}_{i}^{*}|\left|{KG}_{S}\right)},{\langle {R}_{m}\rangle }_{{R}_{g}}, ({UID}_{i}||{SNID}_{j}{)}_{({R}_{m},{ R}_{g},{ KG}_{S})}$}

Using the *FPR* and *NVR* on *BLP*_10_, *SA*_2_ and *SA*_11_, we get *BLP*_12_.

*BLP*_12_: *MD*_i_ $$|\equiv$$ *GW*_k_ $$|\equiv$$ {$${\langle {R}_{g}\rangle }_{h({UID}_{i}|\left|{KG}_{S}\right)}$$,$${\langle {R}_{s}^{*}\rangle }_{{R}_{m}^{*}}$$, ($${UID}_{i}^{*}{)}_{({R}_{g},{ R}_{s}^{*}, { SK}_{G})}$$}

On the other hand, the application of JR on *BLP*_12_ and *SA*_11_ yields *BLP*_13_.

*BLP*_13_: *MD*_i_ $$|\equiv$$ {$${\langle {R}_{g}\rangle }_{h({UID}_{i}|\left|{KG}_{S}\right)}$$,$${\langle {R}_{s}^{*}\rangle }_{{R}_{m}^{*}}$$, ($${UID}_{i}^{*}{)}_{({R}_{g},{ R}_{s}^{*}, { SK}_{G})}$$}

According to *BLP*_13_, the *SKR* is applied to get *BLP*_14_.

*BLP*_14_: *SN*_j_ $|\equiv$ *SN*_j_ $\stackrel{ {SK}_{S}}{\leftrightarrow }$* MD*_i_ and hence security **Goal 1** is achieving.

Based on *BLP*_14_ and *SA*_14_, the *SKR* is applied to obtain *BLP*_15_.

*BLP*_15_: *SN*_j_ $|\equiv$ *MD*_i_ $|\equiv$ *SN*_j_ $\stackrel{ {SK}_{S}}{\leftrightarrow }$* MD*_i_, achieve **Goal 2**.

On the other hand, using *SKR* on *BLP*_14_ yields *BLP*_16_.

*BLP*_16_: *MD*_i_ $|\equiv$ *SN*_j_ $\stackrel{ {SK}_{D}}{\leftrightarrow }$* MD*_i_ and hence **Goal 3** is realized.

The application of *SKR* on *BLP*_14_, *SA*_5_ and *SA*_11_ results in *BLP*_17_.

*BLP*_17_: *MD*_i_ $|\equiv$ *SN*_j_ $|\equiv$ *SN*_j_ $\stackrel{ {SK}_{D}}{\leftrightarrow }$* MD*_i_, attaining security **Goal 4**.

Using idealized form of message *Auth*_2_, the *BR* is applied to get *BLP*_18_.

*BLP*_18_: *GW*_k_ $$\triangleleft$${$${\langle {R}_{s}\rangle }_{{KG}_{S}}$$, ($${R}_{s}{)}_{({SK}_{S},{ KG}_{S})}$$}

However, the usage of MMR on both *BLP*_18_ and *SA*_7_ results in *BLP*_19_.

*BLP*_19_: *GW*_k_ $$|\equiv$$* SN*_j_ ~ {$${\langle {R}_{s}\rangle }_{{KG}_{S}}$$, ($${R}_{s}{)}_{({SK}_{S},{ KG}_{S})}$$}

Based on *BLP*_19_ and *SA*_3_, *NVR* and *FPR* are applied to obtain *BLP*_20_.

*BLP*_20_: *GW*_k_ $$|\equiv$$* SN*_j_ $$|\equiv$$ {$${\langle {R}_{s}\rangle }_{{KG}_{S}}$$, ($${R}_{s}{)}_{({SK}_{S},{ KG}_{S})}$$}

On the other hand, using JR on *BLP*_20_, *SA*_7_ and *SA*_13_ yields *BLP*_21_.

*BLP*_21_: *GW*_k_ $$|\equiv$$ {$${\langle {R}_{s}\rangle }_{{KG}_{S}}$$, ($${R}_{s}{)}_{({SK}_{S},{ KG}_{S})}$$}

However, using the *SKR* on both *BLP*_21_ and *SA*_8_ yields *BLP*_22_.

*BLP*_22_: *GW*_k_ $|\equiv$ *GW*_k_ $\stackrel{ {SK}_{G}}{\leftrightarrow }$ *SN*_j_, realizing security **Goal 7**.

Based on *BLP*_22_, *SA*_13_ and *SA*_15_, the *SKR* is applied to obtain *BLP*_23_.

*BLP*_23_: *GW*_k_ $|\equiv$ *SN*_j_ $|\equiv$ *GW*_k_ $\stackrel{ {SK}_{G}}{\leftrightarrow }$ *SN*_j_ and hence **Goal 8** is attained.

The attainment of all the 8 formulated security goals demonstrates that the proposed scheme achieves strong mutual authentication among the *SN*_j_, *MD*_i_ and *GW*_k_. In addition, it confirms that after successful mutual authentication, session key *SK*_D_ = *SK*_G_ = *SK*_S_ is established among these three entities.

### Informal security analysis

In this sub-section, we state and proof various propositions to show that our scheme supports numerous security features and is robust against many typical smart city attacks. Based on the attack model in “Attack model” section, an adversary is capable of launching attacks such as de-synchronization, denial of service, eavesdropping, session hijacking, KSSTI, replays, forgery, MitM, privileged insider,physical, side-channeling and impersonation. In this sub-section, we demonstrate that our protocol mitigates all these attacks.

#### Proposition 1

*Eavesdropping attacks are prevented.*

#### Proof

Suppose that an adversary *Å* is interested in intercepting the exchanged messages after which parameters such as *SNID*_j_ and *UID*_i_ are retrieved. In our scheme, messages *Log*_Req_ = {*A*_5_,* B*_2_,* B*_3_,* B*_4_,* B*_5_}, *Auth*_1_ = {*C*_1_,* C*_2_,* C*_3_,* C*_4_}, *Auth*_2_ = {*C*_5_,* D*_1_} and *Auth*_3_ = {*D*_2_, *D*_3_, *D*_4_} are exchanged over public channels. Here, *A*_5_ = *R*_n_.*P*,* B*_2_ = *UID*_i_ ⊕ *B*_1_, *B*_3_ = *A*_4_ ⊕ *R*_m_, *B*_4_ = *h* (*UID*_i_||*R*_m_) ⊕ *SNID*_j_, *B*_5_ = *h* (*A*_4_||*SNID*_j_||*B*_1_||*R*_m_), *C*_1_ = *UID*_i_^*^ ⊕ *K*_GS_^*^, *C*_2_ = *R*_g_ ⊕ *h* (*UID*_i_^*^||*K*_GS_^*^), *C*_3_ = *R*_g_ ⊕ *R*_m_^*^, *C*_4_ = *h* (*UID*_i_^*^||*SNID*_j_^*^||*K*_GS_^*^||*R*_m_^*^||*R*_g_),* C*_5_ = *R*_s_ ⊕ *K*_GS_,* D*_1_ = *h* (*K*_GS_||*SK*_S_||*R*_s_), *D*_2_ = *A*_4_^*^ ⊕ *R*_g_, *D*_3_ = *R*_m_^*^ ⊕ *R*_s_^*^ and *D*_4_ = *h* (*UID*_i_^*^||*SK*_G_||*R*_g_||*R*_s_^*^). Clearly, none of these messages contain *SNID*_j_ and *UID*_i_ in plaintext. Therefore, eavesdropping attacks against our scheme fail.

#### Proposition 2

*Our scheme thwarts session hijacking and denial of service attacks.*

#### Proof

The aim of adversary *Å* in this attack is to gain access to the *MD*_i_ belonging to user* U*_i_, effectively disconnecting him/her from accessing sensory data. To prevent this, our scheme incorporates invalid password, identity and biometric checks. For biometric authentication, the the *MD*_i_ checks whether *h* (*CP*_i_^*^) ≟ λ = *h* (*CP*_i_). On the other hand, user password and identity are verified by the *MD*_i_ through the confirmation of whether *A*_2_^*^≟* A*_2_. In both cases, the session is terminated upon validation failure. Therefore, unauthorized logins that can facilitate session hijacking and denial of service attacks are thwarted.

#### Proposition 3

*Message replay and de-synchronization attacks are prevented.*

#### Proof

During the login, authentication and session key negotiation phases, random nonces are incorporated in all the exchanged messages. These random nonces include *R*_m_, *R*_n,_ *R*_g_ and *R*_s_ included in parameters *A*_5_ = *R*_n_.*P*,* B*_1_ = *R*_n_.*P*_k_ = *R*_n_.*nP*, *B*_3_ = *A*_4_ ⊕ *R*_m_, *B*_4_ = *h* (*UID*_i_||*R*_m_) ⊕ *SNID*_j_, *B*_5_ = *h* (*A*_4_||*SNID*_j_||*B*_1_||*R*_m_),* C*_2_ = *R*_g_ ⊕ *h* (*UID*_i_^*^||*K*_GS_^*^), *C*_3_ = *R*_g_ ⊕ *R*_m_^*^, *C*_4_ = *h* (*UID*_i_^*^||*SNID*_j_^*^||*K*_GS_^*^||*R*_m_^*^||*R*_g_),* C*_5_ = *R*_s_ ⊕ *K*_GS_,* D*_1_ = *h* (*K*_GS_||*SK*_S_||*R*_s_), *D*_2_ = *A*_4_^*^ ⊕ *R*_g_, *D*_3_ = *R*_m_^*^ ⊕ *R*_s_^*^ and *D*_4_ = *h* (*UID*_i_^*^||*SK*_G_||*R*_g_||*R*_s_^*^). Therefore, the freshness of messages *Log*_Req_ = {*A*_5_,* B*_2_,* B*_3_,* B*_4_,* B*_5_}, *Auth*_1_ = {*C*_1_,* C*_2_,* C*_3_,* C*_4_}, *Auth*_2_ = {*C*_5_,* D*_1_} and *Auth*_3_ = {*D*_2_, *D*_3_, *D*_4_} is upheld, thwarting any replay attacks. This is in contrast to most schemes that employ timestamps to prevent replay attacks. In these schemes, these timestamps render them vulnerable to de-synchronization attacks.

#### Proposition 4

*Our scheme is robust against privileged insider and impersonation attacks.*

#### Proof

The aim of this attack is to allow users with elevated privileges such as system administrators to access users’ registration information. Thereafter, the obtained information is utilized to impersonate the legitimate users. During the user registration phase, registration request *Req* = {*UID*_i_,* A*_1_,* β*_i_} is constructed by *U*_i_ and forwarded to the *GW*_k_ over secure channels. Here, *UID*_i_ is the user’s unique identity, *β*_i_ is the user’s biometric data and *A*_1_ = *h* (*PW*_i_||*R*_a_). Evidently, privileged users cannot retrieve user’s password *PW*_i_ from *A*_1_ due to its encapsulation in random nonce *R*_a_ and eventual one-way hashing, which is computationally infeasible to reverse.

#### Proposition 5

*Untraceability and anonymity are preserved.*

#### Proof

Suppose that adversary *Å* is interested in tracking particular users and sensors within the network. To realize this, all the messages exchanged over the public channels are intercepted. These messages include *Log*_Req_ = {*A*_5_,* B*_2_,* B*_3_,* B*_4_,* B*_5_}, *Auth*_1_ = {*C*_1_,* C*_2_,* C*_3_,* C*_4_}, *Auth*_2_ = {*C*_5_,* D*_1_} and *Auth*_3_ = {*D*_2_, *D*_3_, *D*_4_}. Thereafter, attempts are made to obtain *SNID*_j_ and *UID*_i_. However, according to *Proposition 1*, this attempt will fail. Although parameters *C*_2_ = *R*_g_ ⊕ *h* (*UID*_i_^*^||*K*_GS_^*^),* C*_4_ = *h* (*UID*_i_^*^||*SNID*_j_^*^||*K*_GS_^*^||*R*_m_^*^||*R*_g_), and *D*_4_ = *h* (*UID*_i_^*^||*SK*_G_||*R*_g_||*R*_s_^*^) contain these unique identities, they are scrambled in other security tokens and hashed. This makes it cumbersome for adversary *Å* to retrieve them. To prevent traceability attacks, the *MD*_i_ generates random nonces *R*_a_,* R*_m_ and *R*_n_ that are incorporated in values* A*_5_ = *R*_n_.*P*, *B*_1_ = *R*_n_.*P*_k_, *B*_3_ = *A*_4_ ⊕ *R*_m_, *B*_4_ = *h* (*UID*_i_||*R*_m_) ⊕ *SNID*_j_ and *B*_5_ = *h* (*A*_4_||*SNID*_j_||*B*_1_||*R*_m_). Similarly, the *SN*_j_ generates nonce *R*_s_ that is incorporated in parameters *C*_5_ = *R*_s_ ⊕ *K*_GS_, session key *SK*_S_ = *h* (*UID*_i_^*^||*SNID*_j_^*^||*R*_m_^*^||*R*_g_^*^||*R*_s_) and value *D*_1_ = *h* (*K*_GS_||*SK*_S_||*R*_s_). Therefore, user’s login request message *Log*_Req_ and *SN*_j_’s authentication message *Auth*_2_ are session-specific. As such, it is difficult for the adversary to associate these two messages to particular users and sensors.

#### Proposition 6

*Our scheme is resilient against side-channeling and physical attacks.*

#### Proof

The goal of the attacker is to steal user’s *MD*_i_ and use power analysis techniques to retrieve the stored secrets. In our scheme, the *MD*_i_ stores value set {*f* (.), λ,* ε*,* A*_2_,* A*_3_,* P*_k_,* R*_a_} in its memory. Here, λ = *h* (*CP*_i_), *ε* = *CP*_i_ ⊕ *β*_i_,* A*_1_ = *h* (*PW*_i_||*R*_a_),* A*_2_ = *h* (*UID*_i_||*A*_1_||*CP*_i_), *A*_3_ = *h* (*UID*_i_||*M*_k_) ⊕ *h* (*A*_1_||*CP*_i_), *CP*_i_ is the code-phrase chosen by the *GW*_k_, *R*_a_ is the random nonce generated by the *MD*_i_ while *P*_k_ = *nP* is the public key computed at the *GW*_k_. Next, an attempt is made to retrieve user’s unique identity *UID*_i_ and password *PW*_i_. This requires access to security tokens such as *CP*_i_ and master key *M*_k_ for *GW*_k_. In addition, adversary *Å* needs to reverse the one-way hashing function to obtain these parameters from *A*_1_and* A*_2_. Since this presents a computationally infeasible activity, this attack flops.

#### Proposition 7

*Known Session-Specific Temporary Information (KSSTI) attacks are prevented.*

#### Proof

In our scheme, all the three entities derive the session key used to encipher the sensory data. Whereas the *SN*_j_ derives the session key as *SK*_S_ = *h* (*UID*_i_^*^||*SNID*_j_^*^||*R*_m_^*^||*R*_g_^*^||*R*_s_), the *GW*_k_ derives it as *SK*_G_ = *h* (*UID*_i_^*^||*SNID*_j_^*^||*R*_m_^*^||*R*_g_||*R*_s_^*^). Similarly, the *MD*_i_ computes the session key as *SK*_D_ = *h* (*UID*_i_||*SNID*_j_||*R*_m_||*R*_g_^*^||*R*_s_^*^). Based on *Propositions 1* and *5*, adversary cannot obtain identities *UID*_i_ and *SNID*_j_ from the exchanged messages. In addition, *Proposition 6* has detailed the difficulty of obtaining *UID*_i_ from *MD*_i_’s memory. Therefore, even if temporary information such as random nonces *R*_m_, *R*_g_ and *R*_s_ are compromised by *Å*, these session keys cannot be computed.

#### Proposition 8

*Strong mutual authentication is executed among all network entities.*

#### Proof

In our scheme, the *MD*_i_ validates user biometric data by checking whether *h* (*CP*_i_^*^) ≟ λ = *h* (*CP*_i_). In addition, it verifies user unique identity *UID*_i_ and password *PW*_i_ by confirming if *A*_2_^*^≟* A*_2_. On its part, the the *GW*_k_ authenticates *MD*_i_ by checking whether *B*_5_^*^≟* B*_5_, while the *SN*_j_ validates *GW*_k_ through the confirmation of whether *D*_1_^*^≟* D*_1_. Finally, the the *MD*_i_ authenticates the *SN*_j_ by establishing whether *D*_4_^*^≟* D*_4_. In all these authentication scenarios, the session is aborted upon validation failure.

#### Proposition 9

*Session keys are negotiated among all network entities.*

#### Proof

To protect the exchanged sensor data, the *MD*_i_, *GW*_k_ and *SN*_j_ setup session keys amongst themselves. Upon receiving authentication message *Auth*_1_ = {*C*_1_,* C*_2_,* C*_3_,* C*_4_}, the *SN*_j_ computes values *UID*_i_^*^ = *C*_1_ ⊕ *K*_GS_^*^,* R*_g_^*^ = *C*_2_ ⊕ *h* (*UID*_i_^*^||*K*_GS_^*^), *R*_m_^*^ = *R*_g_^*^ ⊕ *C*_3_, *C*_4_^*^ = *h* (*UID*_i_^*^||*SNID*_j_^*^||*K*_GS_||*R*_m_^*^||*R*_g_^*^),* C*_5_ = *R*_s_ ⊕ *K*_GS_ and session key *SK*_S_ = *h* (*UID*_i_^*^||*SNID*_j_^*^||*R*_m_^*^||*R*_g_^*^||*R*_s_). Similarly, on getting authentication response message *Auth*_2_ = {*C*_5_,* D*_1_}, the *GW*_k_ derives value* R*_s_^*^ = *C*_5_ ⊕ *K*_GS_^*^ and session key *SK*_G_ = *h* (*UID*_i_^*^||*SNID*_j_^*^||*R*_m_^*^||*R*_g_||*R*_s_^*^). On its part, the *MD*_i_ receives authentication message *Auth*_3_ = {*D*_2_, *D*_3_, *D*_4_} after which it derives values *R*_g_^*^ = *A*_4_ ⊕ *D*_2_, *R*_s_^*^ = *R*_m_ ⊕ *D*_3_ and session key *SK*_D_ = *h* (*UID*_i_||*SNID*_j_||*R*_m_||*R*_g_^*^||*R*_s_^*^). These session keys are used by these entities to encipher the sensor data exchanged between the *MD*_i_ and *SN*_j_ via the *GW*_k_.

#### Proposition 10

*Our scheme is robust against MitM and forgery attacks.*

#### Proof

The aim of adversary *Å* is to gather information belonging to the network entities and attempt to forge the exchanged messages *Log*_Req_ = {*A*_5_,* B*_2_,* B*_3_,* B*_4_,* B*_5_}, *Auth*_1_ = {*C*_1_,* C*_2_,* C*_3_,* C*_4_}, *Auth*_2_ = {*C*_5_,* D*_1_} and *Auth*_3_ = {*D*_2_, *D*_3_, *D*_4_}. Here, *A*_1_ = *h* (*PW*_i_||*R*_a_), *A*_3_ = *h* (*UID*_i_||*M*_k_) ⊕ *h* (*A*_1_||*CP*_i_), *A*_4_ = *A*_3_ ⊕ *h* (*h(PW*_i_||*R*_a_)||*CP*_i_^*^), *A*_5_ = *R*_n_.*P*,* B*_1_ = *R*_n_.*P*_k_ = *R*_n_.*nP*, *B*_2_ = *UID*_i_ ⊕ *B*_1_, *B*_3_ = *A*_4_ ⊕ *R*_m_, *B*_4_ = *h* (*UID*_i_||*R*_m_) ⊕ *SNID*_j_, *B*_5_ = *h* (*A*_4_||*SNID*_j_||*B*_1_||*R*_m_),* C*_1_ = *UID*_i_^*^ ⊕ *K*_GS_^*^, *C*_2_ = *R*_g_ ⊕ *h* (*UID*_i_^*^||*K*_GS_^*^), *C*_3_ = *R*_g_ ⊕ *R*_m_^*^, *C*_4_ = *h* (*UID*_i_^*^||*SNID*_j_^*^||*K*_GS_^*^||*R*_m_^*^||*R*_g_),* C*_5_ = *R*_s_ ⊕ *K*_GS_,* D*_1_ = *h* (*K*_GS_||*SK*_S_||*R*_s_), *D*_2_ = *A*_4_^*^ ⊕ *R*_g_, *D*_3_ = *R*_m_^*^ ⊕ *R*_s_^*^ and *D*_4_ = *h* (*UID*_i_^*^||*SK*_G_||*R*_g_||*R*_s_^*^). To forge these messages, *Å* needs access to *GW*_k_’s master key *P*_k_, *UID*_i_, *SNID*_j_, *PW*_i_, *CP*_i_^*^, *M*_k_, *SK*_S_, *SK*_G_, *K*_GS_ as well as random nonces *R*_a_, *R*_g_, *R*_m_ , *R*_n_ and *R*_s_. *Proposition 1* , *Proposition 5* and *Proposition 6* have demonstrated the difficulty that *Å* faces in obtaining *UID*_i_ and *SNID*_j_. On the other hand, *Propositions 4 and 6* have shown the challenges *Å* faces in retrieving *PW*_i_. Similarly, *Proposition 7* has demonstrated the diffulty of adversarial derivation of session keys *SK*_S_, *SK*_G_ and *SK*_D_. Since *M*_k_ is only known to *GW*_k_ and *K*_GS_ is only known by *GW*_k_ and *SN*_j_,* Å* cannot access these values. Similarly, random nonces are independently derived at the *MD*_i_, *GW*_k_ and *SN*_j_, hence not available to *Å*. As such, forgery attacks against our scheme flops.

#### Proposition 11

*Backward and forward key secrecy is upheld.*

#### Proof

In our scheme, the *SN*_j_ computes session key as *SK*_S_ = *h* (*UID*_i_^*^||*SNID*_j_^*^||*R*_m_^*^||*R*_g_^*^||*R*_s_) while the *GW*_k_ derives the session key as *SK*_G_ = *h* (*UID*_i_^*^||*SNID*_j_^*^||*R*_m_^*^||*R*_g_||*R*_s_^*^). Similarly, the *MD*_i_ calculates the session key as *SK*_D_ = *h* (*UID*_i_||*SNID*_j_||*R*_m_||*R*_g_^*^||*R*_s_^*^). The incorporation of random nonces *R*_m_, *R*_g_^*^ *R*_s_^*^ renders the derived session keys one-time such that they are only valid for a particular session. Therefore, although adversary *Å* compromises the current session keys, it is not possible to use the captured parameters to derive session keys for the previous and subsequent communication session.

## Performance evaluation

In this section, we present the comparative evaluations of our scheme in terms of computation costs, communication costs, functional and security features. The specific details are elaborated in the sub-sections below.

### Computation costs

The proposed scheme is implemented in a laptop with the specifications in Table 2. Using the specifications in Table 2, the execution time times for the the elliptic curve point multiplication (*T*_EM_) ≈ 21.74 ms, one-way hashing (*T*_H_) ≈ 0.63 ms and elliptic curve point addition (*T*_EA_) ≈ 6.75 ms.

**Table 2:** Implementation environment.

Specification	Details
Operating system	Windows 11 Pro 64-bit
Processor	Intel Core i5-10400
Clock speed	2.90 GHz
RAM	8 GB
Programming language	Python
Cryptographic library	Pycryptodome

During the login, authentication and key negotiation phase, the *MD*_i_ executes 2 ECC point multiplications and 8 one-way hashing operations. On the other hand, the *GW*_k_ carries out a single ECC point multiplication and 9 one-way hashing operations. On its part, the *SN*_j_ executes only 4 one-way hashing operations. Therefore, the total computation cost of our scheme is 21*T*_H_ + 3 *T*_EM_. Table 3 presents the computation costs comparative evaluation of our scheme against other related schemes.

**Table 3:** Computation costs comparisons.

Scheme	Time (ms)
Li et al.^31^	24*T*_H_ + 6*T*_EM_ ≈ 145.56
Kumar et al.^61^	5*T*_H_ + 6 *T*_EM_ ≈ 133.59
Nikooghadam et al.^68^	19*T*_H_ + 4 *T*_EM_ ≈ 98.93
Wang et al.^71^	11*T*_H_ + 10*T*_EM_ + 4*T*_EA_ ≈ 251.33
Bera et al.^72^	18*T*_H_ + 10*T*_EM_ + 3*T*_EA_ ≈ 248.99
Bagga et al.^73^	10*T*_H_ + 9*T*_EM_ + 2*T*_EA_ ≈ 215.46
Proposed	21*T*_H_ + 3 *T*_EM_ ≈ 78.45

**Figure 4:**
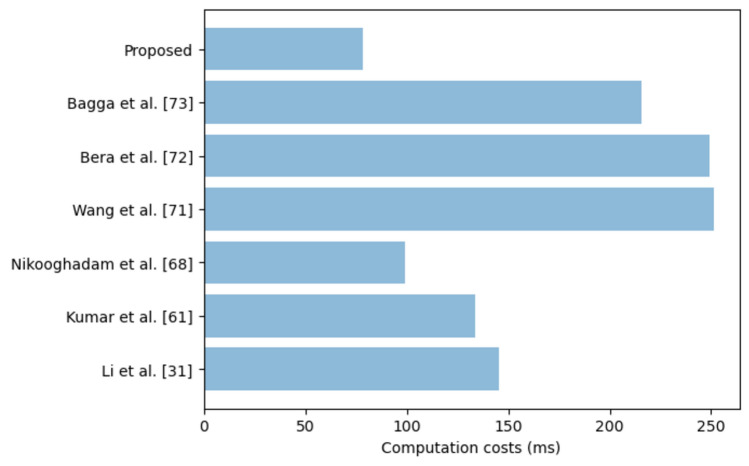
Computation costs comparisons.

As shown in Fig. 4, the scheme developed in^71^ incurs the highest computation costs of 251.33 ms. This is attributed to the numerous elliptic curve point multiplications which are computationally intensive. This is followed by the protocols in^31,61,68,72,73^ which incur computation overheads of 248.99 ms, 215.46 ms, 145.56 ms, 133.59 ms and 98.93 ms respectively.

On the other hand, the proposed scheme incurs the lowest computation costs of only 78.45 ms. Based on the scheme in^68^, our protocol reduced the computation costs by 20.7%. Since the sensors in smart cities are limited in terms of the computation power, our scheme is the most ideal for deployment in this environment.

### Communication costs

In the course of the login, authentication and session key setup phase, 4 messages are exchanged among the *MD*_i_, *GW*_k_ and *SN*_j_. These messages include *Log*_Req_ = {*A*_5_,* B*_2_,* B*_3_,* B*_4_,* B*_5_}, *Auth*_1_ = {*C*_1_,* C*_2_,* C*_3_,* C*_4_}, *Auth*_2_ = {*C*_5_,* D*_1_} and *Auth*_3_ = {*D*_2_, *D*_3_, *D*_4_}. Here, ECC point multiplication = 160 bits, identities = 32 bits, one way hashing = 160 bits and random nonces = 128 bits. Using these values, *Log*_Req_ = 160 + 160 + 160 + 160 + 160 = 800 bits, *Auth*_1_ = 160 + 160 + 128 + 160 = 608 bits, *Auth*_2_ = 160 + 160 = 320 bits and *Auth*_3_ = 160 + 128 + 160 = 448 bits. As such, the total communication overhead is 2176 bits. Table 4 provides comparative evaluation of the communication costs of our scheme against other related protocols.

**Table 4:** Communication costs comparisons.

Scheme	Size (bits)
Li et al.^31^	1792
Kumar et al.^61^	1760
Nikooghadam et al.^68^	2336
Wang et al.^71^	1376
Bera et al.^72^	1952
Bagga et al.^73^	1856
Proposed	2176

**Figure 5:**
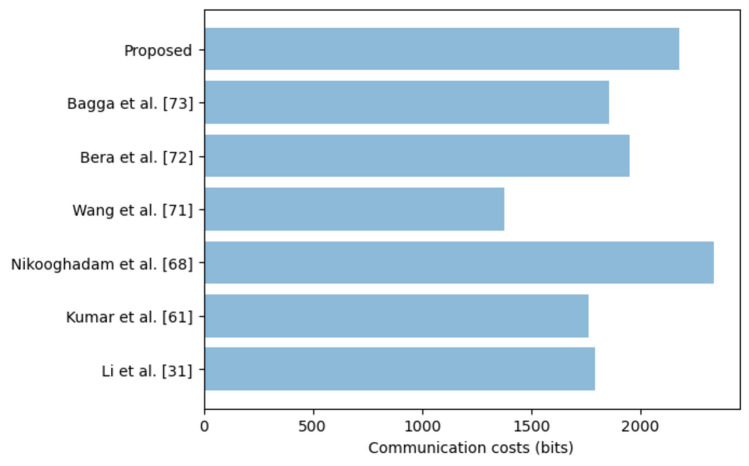
Communication costs comparisons.

As shown in Fig. 5, the protocol in^68^ has the highest communication costs of 2336 bits. This is followed by the proposed scheme which inclurs a communication overhead of 2176 bits. This is attributed to the strong mutual authentication that must be executed among the *MD*_i_, *GW*_k_ and *SN*_j_.

Although the protocols in^31,61,71–73^ incur relatively lower communication costs, they are insecure since they cannot offer functional and security features supported by our scheme, as evidenced in Table 5.

**Table 5:** Functional and security features.

	^72^	^71^	^73^	^31^	^61^	^68^	Proposed
*Security features*							
Mutual authentication	√	√	√	√	√	×	√
Key agreement	√	√	√	√	√	√	√
Backward key secrecy	×	√	√	×	√	√	√
Forward key secrecy	×	√	√	×	√	√	√
Anonymity	√	√	×	√	√	√	√
Untraceability	√	√	×	√	×	√	√
Password change	×	√	×	√	×	×	√
Formal verification	√	√	√	√	√	√	√
*Resilient against*							
De-synchronization	×	×	×	×	×	×	√
Denial of service	×	×	×	√	√	×	√
Eavesdropping	×	×	×	×	×	×	√
Session hijacking	×	×	×	×	×	×	√
KSSTI	×	×	×	×	√	×	√
Replays	√	√	√	×	×	×	√
Forgery	×	×	×	×	×	×	√
MitM	√	×	√	×	√	×	√
Privileged insider	√	×	√	×	√	×	√
Physical	√	×	√	√	√	√	√
Side-channeling	×	×	×	×	×	×	√
Impersonation	√	√	√	×	√	×	√
	√ Supported × Not supported or not considered						

### Functional and security features

In this sub-section, we discusses the comparative evaluation of our scheme in terms of offered functional and security features. Table 5 presents the security features supported by our scheme as well as the attacks that this scheme is resilient against. The security features and resilience of its peers are also detailed.

As shown in Table 5, the protocol in^68^ supports only 7 functionalities and hence is the most insecure. This is followed by the scheme in^31^ which supports 8 security features. On the other hand, the protocols in^71–73^ support 10 functionalities each. However, the protocol developed in^61^ supports 12 functionalities while the proposed scheme offers support for all the 20 security features and functionalities. Although our scheme incurs slightly higher communication overheads, it supports the highets number of security and privacy functionalites. In addition, it incurs the lowest computation costs. As such, it offers a good trade-off between privacy, security and performance.

Some of the anticipated limitations that are likely to crop up during the practical implementation of our scheme is its slightly high communication costs and the need for biometric reader at the user mobile device *MD*_i_. Specifically, the accurate recovery of biometric tokens via fuzzy extraction is not a trivial exercise.

## Conclusion and future work

The security, privacy and performance issues in smart cities have attracted a lot of attention from the industry and academia. Therefore, past research works have developed a myriad of security solutions for this environment. In majority of these approaches, public key cryptography, blockchain and bilinear pairing operations are utilized. As such, the resulting authentication process is computationally extensive and hence long latencies can be experienced. In addition, they place high communication, energy and storage overheads on the resource-limited smart city sensor devices. Motivated by this, we have presented a biometric-based scheme that has been demonstrated to incur the least computation overheads. Its formal security analysis has shown that it performs strong mutual authentication and key negotiation in an appropriate manner. In addition, informal security analysis has shown that it is secure under all the threat assumptions in the Canetti and Krawczyk attack model. Future research work will involve further reductions in the communication overheads which are observed to be slightly higher compared with some of its peers.

## Data Availability

The datasets generated and/or analyzed during the current study are not publicly available due to university policy but are available from the corresponding author on reasonable request.
